# A review of visual SLAM for robotics: evolution, properties, and future applications

**DOI:** 10.3389/frobt.2024.1347985

**Published:** 2024-04-10

**Authors:** Basheer Al-Tawil, Thorsten Hempel, Ahmed Abdelrahman, Ayoub Al-Hamadi

**Affiliations:** Institute for Information Technology and Communications, Otto-von-Guericke-University, Magdeburg, Germany

**Keywords:** V-SLAM, interactive mobile robots, ROS, benchmark, Middleware, workflow, robotic applications, robotic ecosystem

## Abstract

Visual simultaneous localization and mapping (V-SLAM) plays a crucial role in the field of robotic systems, especially for interactive and collaborative mobile robots. The growing reliance on robotics has increased complexity in task execution in real-world applications. Consequently, several types of V-SLAM methods have been revealed to facilitate and streamline the functions of robots. This work aims to showcase the latest V-SLAM methodologies, offering clear selection criteria for researchers and developers to choose the right approach for their robotic applications. It chronologically presents the evolution of SLAM methods, highlighting key principles and providing comparative analyses between them. The paper focuses on the integration of the robotic ecosystem with a robot operating system (ROS) as Middleware, explores essential V-SLAM benchmark datasets, and presents demonstrative figures for each method’s workflow.

## 1 Introduction

Robotics is an interdisciplinary field that involves the creation, design, and operation of tasks using algorithms and programming (Bongard, 2008; Joo et al., 2020; Awais and Henrich 2010; Fong et al., 2003). Its impact extends to manufacturing, automation, optimization, transportation, medical applications, and even NASA’s interplanetary exploration (Li et al., 2023b; Heyer, 2010; Sheridan, 2016; Mazumdar et al., 2023). Service robots, which interact with people, are becoming more common and useful in everyday life (Hempel et al., 2023; Lynch et al., 2023). The imperative of integrating automation with human cognitive abilities becomes evident in facilitating a successful collaboration between humans and robots. This helps service robots be more effective in different situations where they interact with people (Prati et al., 2021; Strazdas et al., 2020; Zheng et al., 2023). Furthermore, using multiple robots together can help them handle complex tasks better (Zheng et al., 2022; Li et al., 2023b; Fiedler et al., 2021). To manage and coordinate various processes, a robot operating system (ROS) plays a significant role (Buyval et al., 2017). It is an open-source framework that aids roboticists in implementing their research and projects with minimal complexity. ROS offers a multitude of features, including hardware integration, control mechanisms, and seamless device implementation into the system, thus facilitating the development and operation of robotic systems (Altawil and Can 2023).

**FIGURE 1:**
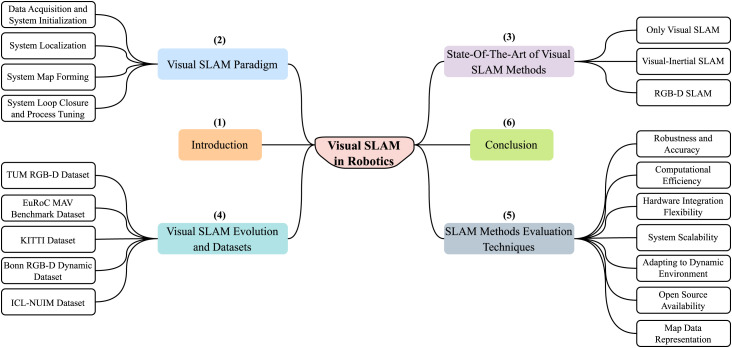
Article organizational chart.

As shown in Figure 1, the paper is divided into six sections. Section 1 gives the brief introduction about robotics and SLAM. Section 2 presents an overview of the V-SLAM paradigm that delves into its fundamental concepts.

Section 3 presents the state-of-the-art V-SLAM methods, offering insights into the latest advancements of them. Moving forward, section 4 explores the evolution of V-SLAM and discusses the most commonly used datasets. Section 5 focuses on techniques for evaluating SLAM methods, aiding in the selection of appropriate methods. Finally, Section 6 provides the conclusion of the article, summarizing the key points we discovered while working on our review paper. 

 Recently, we require robots that can move around and work well in places they have never been before. In this regard, simultaneous localization and mapping (SLAM) emerges as a fundamental approach for these robots. The primary goal of SLAM is to autonomously explore and navigate unknown environments by simultaneously creating a map and determining their own position (Durrant-Whyte, 2012; Mohamed et al., 2008). Furthermore, it provides real-time capabilities, allowing robots to make decisions on-the-fly without relying on pre-existing maps. Its utility extends to the extraction, organization, and comprehension of information, thereby enhancing the robot’s capacity to interpret and interact effectively with its environment (Pal et al., 2022; Lee et al., 2020; Aslan et al., 2021). It is crucial to enable these robots to autonomously navigate and interact in human environments, thus reducing human effort and enhancing overall productivity (Arfa, 2022). The construction of maps is based on the utilization of sensor data, such as visual data, laser scanning data, and data from the inertial measurement unit (IMU), followed by rapid processing (Macario Barros et al., 2022).

Historically, prior to the advent of SLAM technology, localization and mapping were treated as distinct entities. However, it was seen that there is a strong internal dependency between mapping and localization. Although accurate localization depends on the map, mapping depends on localization. Thus, the question is known as the “Chicken and Egg” question (Taheri and Xia, 2021). In robotics, there are different tools to help robots obtain information from surroundings and build their map. One way is to use sensors such as LiDAR, which uses light detection and ranging sensors to make a 3D map (Huang, 2021; Van Nam and Gon-Woo, 2021). Another way is to use cameras, such as monocular and stereo cameras, which are applied in visual SLAM (V-SLAM). In this method, the robot uses pictures to figure out where it is and creates the required map (Davison et al., 2007). Regarding the paper’s intensive details, we provide Table 1 that summarizes and includes the description of abbreviations used in the article based on SLAM principles and fundamentals.

**TABLE 1:** List of abbreviations used in this article.

Abbreviation	Explanation	Abbreviation	Explanation
V-SLAM	Visual simultaneous localization and mapping	LSD	Large-scale direct
ROS	Robot Operating System	OKVIS	Open keyframe-based visual–inertial
Lidar	Light detection and ranging	DVO	Dense visual odometry
BA	Bundle adjustment	RPGO	Robust pose-graph optimization
BoW	Bag of words	IMU	Inertial measurement unit
PTAM	Parallel tracking and mapping	GPS	Global positioning system
FAST	Features from accelerated segment test	MAV	Micro air vehicle
ROVIO	Robust visual–inertial odometry	AGV	Automated-guided vehicle
HRI	Human–robot interaction	UAV	Unmanned aerial vehicle
DTAM	Dense tracking and mapping	AR	Augmented reality
LCP	Loop closure process	VR	Virtual reality
SS	Semantic segmentation	RoLi	Range of light intensity
DSt	Dense stereo	ILR	Illumination and light robustness
DSe	Dense semantics	BRIEF	Binary Robust Independent Elementary Features
SCE	Spatial coordinate errors		

**FIGURE 2:**
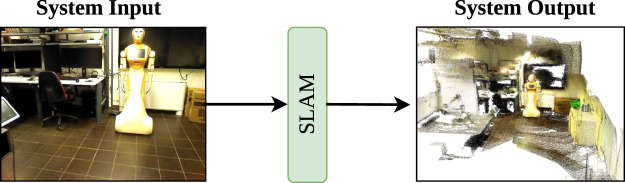
Schematic representation of a robotic system’s architecture, highlighting the incorporation of SLAM and its location within the system.

Due to the significance of visual techniques in interactive robotic applications, our research focuses on V-SLAM methodologies and their evaluation. V-SLAM can be applied to mobile robotics that utilizes cameras to create a map of their surroundings and easily locate themselves within their work space (Li et al., 2020). It uses techniques such as computer vision to extract and match visual data for localization and mapping (Zhang et al., 2020; Chung et al., 2023). It allows robots to map complex environments while performing tasks such as navigation in dynamic fields (Placed et al., 2023; Khoyani and Amini 2023). It places a strong emphasis on accurate tracking of camera poses and estimating past trajectories of the robot during its work (Nguyen et al., 2022; Awais and Henrich 2010). Figure 2 provides a basic understanding of V-SLAM. It takes an image from the environment as an input, processes it, and produces a map as an output. In V-SLAM, various types of cameras are used to capture images or videos. A commonly used camera is the monocular camera, which has a single lens, providing 2D visual information (Civera et al., 2011). However, due to its limitation of lacking depth information, researchers often turn to stereo cameras, which are equipped with two lenses set at a specific distance to capture images from different perspectives, enabling depth details (Gao et al., 2020; Meng et al., 2018). Another valuable option in V-SLAM is the use of RGB-D cameras, which are capable of capturing both color information (RGB) and depth information (D) (Meng et al., 2018). Although monocular cameras are inexpensive and lightweight, they may require additional sensors in order to provide accurate data. In contrast, RGB-D and stereo cameras provide depth information. This makes RGB-D, such as Microsoft’s Kinect and stereo cameras, suitable for robust and accurate SLAM systems (Luo et al., 2021).

Previous research demonstrated the effectiveness of V-SLAM methods, but they are often explained with very few details and separate figures (Khoyani and Amini, 2023; Fan et al., 2020), making it challenging to understand, compare, and make selections among them. As a result, our study focuses on simplifying the explanation of V-SLAM methodologies to enable readers to comprehend them easily. The main contributions of the study can be described as follows:

• Investigation into V-SLAM techniques to determine the most appropriate tools for use in robotics.
• Creation of a graphical and illustrative structural workflow for each method to enhance the comprehension of the operational processes involved in V-SLAM.
• Presentation of significant factors for the evaluation and selection criteria among the V-SLAM methods.
• Compilation of a comparative table that lists essential parameters and features for each V-SLAM method.
• Presentation and discussion of relevant datasets employed within the domain of robotics applications.

## 2 Visual SLAM paradigm

As discussed in Introduction, V-SLAM uses sensor data to provide valuable information to the system (Khoyani and Amini, 2023). Mobile robots and autonomous vehicles require the ability to understand their environment to complete their tasks and achieve their goals (Ai et al., 2021). This understanding is essential for them to be successful in their operations (Bongard, 2008).

**FIGURE 3:**
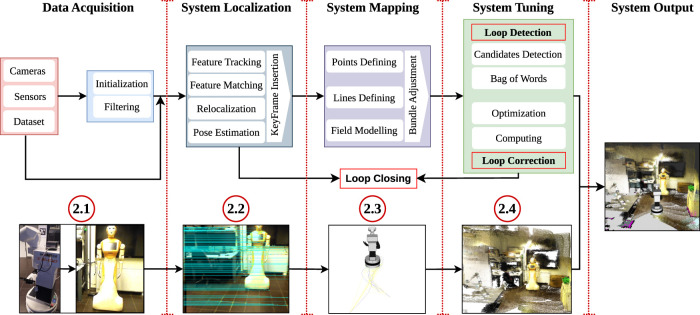
Visual SLAM architecture: an overview of the four core components necessary for visual SLAM: data acquisition, system localization, system mapping, and system loop closure, and process tuning, enabling mobile robots to perceive, navigate, and interact with their environment.

The V-SLAM framework is composed of sequential steps that are organized to create the system and process its data; see Figure 3, which explains the processes performed within V-SLAM in parallel with the demonstrated pictures. This includes the creation of a detailed map, a trajectory estimator, and the precise positioning and orientation of the cameras attached to that system (Beghdadi and Mallem, 2022; Kazerouni et al., 2022). Within this framework, various scenarios can be effectively implemented and operated, such as pixel-wise motion segmentation (Hempel and Al-Hamadi, 2020), semantic segmentation (Liu and Miura, 2021), and filtering techniques (Wang et al., 2023; Grisetti et al., 2007). These approaches aim to achieve a professional approach for a visual representation of the processes involved in V-SLAM. The operational framework has been systematically divided into four sections, which can be listed and explained herein.

### 2.1 Data acquisition and system initialization

In this stage of V-SLAM, we systematically prepare input data using system hardware, which includes capturing and preparing images. It involves installing cameras such as RGB-D cameras, depth cameras, or infrared sensors for collecting data and initializing the system (Beghdadi and Mallem, 2022).

The system gathers data, with a particular emphasis on crucial filtering details aimed at effectively eliminating any noise present in the input data (Mane et al., 2016; Grisetti et al., 2007). The refined data are then sent to the next stage for further processing to extract features from the input information (Ai et al., 2021). As a result, progress in SLAM methods has resulted in the creation of numerous datasets accessible to researchers to evaluate V-SLAM algorithms (El Bouazzaoui et al., 2021).

### 2.2 System localization

In the second stage of V-SLAM, the system focuses on finding its location, which is an important part of the entire process (Scaradozzi et al., 2018). It involves the execution of various processes that are crucial for successfully determining where the robot is. Feature tracking plays a central role during this phase, with a primary focus on tasks such as feature extraction, matching, re-localization, and pose estimation (Picard et al., 2023). It aims to align and identify the frames that guide the estimation and creation of the initial keyframe for the input data (Ai et al., 2021). A keyframe is a set of video frames that includes a group of observed feature points and the camera’s poses. It plays an important role for the tracking and localization process, helping in eliminating drift errors for camera poses attached to the robot (Sheng et al., 2019; Hsiao et al., 2017). Subsequently, this keyframe is sent for further processing in the next stage, where it will be shaped into a preliminary map, a crucial part for the third stage of the workflow (Aloui et al., 2022; Zhang et al., 2020).

### 2.3 System map formation

The third stage of the V-SLAM workflow focuses on the crucial task of building the map, an essential element in V-SLAM processes. Various types of maps can be generated using SLAM, including topological maps, volumetric (3D) maps, such as point cloud and occupancy grid maps, and feature-based or landmark maps. The choice of the map type is based on factors such as the sensors employed, application requirements, environmental assumptions, and the type of dataset used in robotic applications (Taheri and Xia, 2021; Fernández-Moral et al., 2013). In robotics, a grid map is a representation of a physical environment, with each cell representing a particular location and storing data comprising obstacles, topography, and occupancy. It functions as a fundamental data structure for several robotics navigation and localization techniques (Grisetti et al., 2007). A feature-based map is a representation which captures the features of the environment, such as landmarks or objects, to facilitate localization and navigation tasks (Li et al., 2022a). A point cloud map is a representation of a physical space or object made from lots of 3D dots, showing how things are arranged in a place. It is created using special cameras or sensors and helps robots and computers understand what is around them (Chu et al., 2018).

After setting up keyframes during the localization stage, the workflow progresses to field modeling. Then, key points and feature lines are identified and detected, which is crucial for generating a map (Schneider et al., 2018). It is a process that builds and updates the map of an unknown environment and is used to continuously track the robot’s location (Chen et al., 2020). It is a two-way process that works together with the localization process, where they depend on each other to achieve SLAM processes. It gathers real-time data about the surroundings, creating both a geometric and a visual model r13 (accessed on 14 November 2023). In addition, the process includes the implementation of bundle adjustments (BAs) to improve the precision of the generated map before it is moved to the final stage (Acosta-Amaya et al., 2023). BA is a tool that simultaneously refines the parameters essential for estimating and reconstructing the location of observed points in available images. It plays a crucial role in feature-based SLAM (Bustos et al., 2019; Eudes et al., 2010).

### 2.4 System loop closure and process tuning

The final stage in the V-SLAM workflow involves fine-tuning the process and closing loops, resulting in the optimization of the final map. In V-SLAM, the loop closure procedure examines and maintains previously visited places, fixing any errors that might have occurred during the robot’s exploration within an unknown environment. These errors typically result from the estimation processes performed in earlier stages of the SLAM workflow (Tsintotas et al., 2022; Hess et al., 2016). Loop closure and process tuning can be done using different techniques, such as the extended Kalman filter SLAM (EKF-SLAM). EKF-SLAM combines loop closure and landmark observation data to adjust the map in the Kalman filter’s state estimate. This tool helps address uncertainties in the surrounding world (map) and localize the robot within it (Song et al., 2021; Ullah et al., 2020).

The bag-of-words (BoW) approach is another technique used to enable robots to recognize and recall previously visited locations. This is similar to how humans remember places they have been to in the past, even after a long time, due to the activities that took place there. BoW works by taking the visual features of each image and converting them into a histogram of visual words. This histogram is then used to create a fixed-size vector representation of the BoW, which is stored for use in matching and loop-closing processes (Cui et al., 2022; Tsintotas et al., 2022).

Finally, graph optimization is used as a correction tool for loop closure processes. It refines the final map and robot’s trajectory by optimizing the graph based on landmarks. This technique involves a graph-based representation of the SLAM issue, where vertices represent robot poses and map characteristics and edges represent constraints or measurements between the poses. It is commonly used as a correction tool in graph-based SLAM types (Zhang et al., 2017; Chou et al., 2019; Meng et al., 2022).

In conclusion, these comprehensive workflow processes outlined in Sections 2.1, 2.2, 2.3, and 2.4, respectively, play an important role in V-SLAM for robotics as they facilitate the simultaneous creation of maps and real-time location tracking within the operational environment (Li et al., 2022b).

## 3 State-of-the-art of visual SLAM methods

V-SLAM plays a significant role as a transformative topic within the robotics industry and research (Khoyani and Amini, 2023; Acosta-Amaya et al., 2023). The progress in this field can be attributed to tools such as machine learning, computer vision, deep learning, and state-of-the-art sensor technologies, which have collectively simplified and enhanced its strategy in real-life applications (Beghdadi and Mallem, 2022; Duan et al., 2019).

**FIGURE 4:**
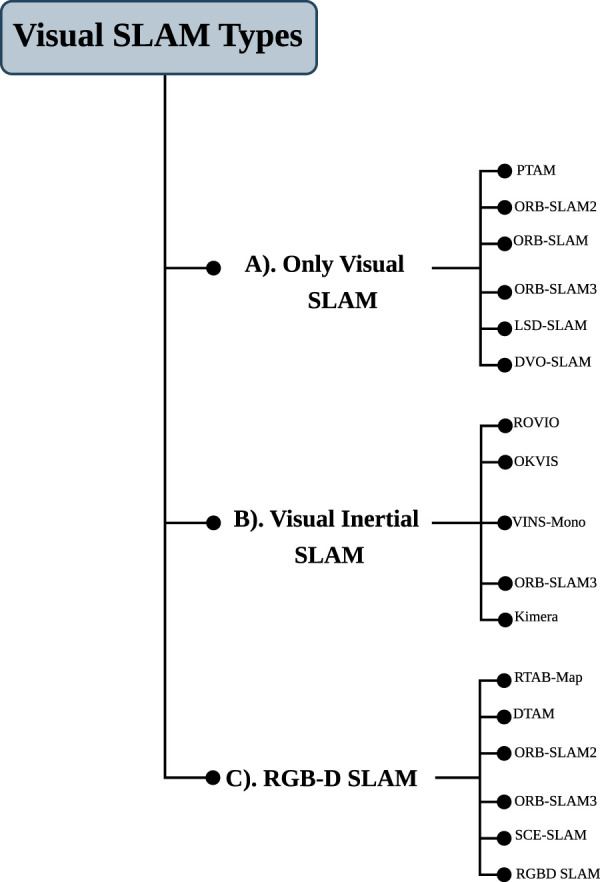
Illustration of visual SLAM types: only-visual SLAM, visual-inertial SLAM, and RGB-D SLAM.

**FIGURE 5:**
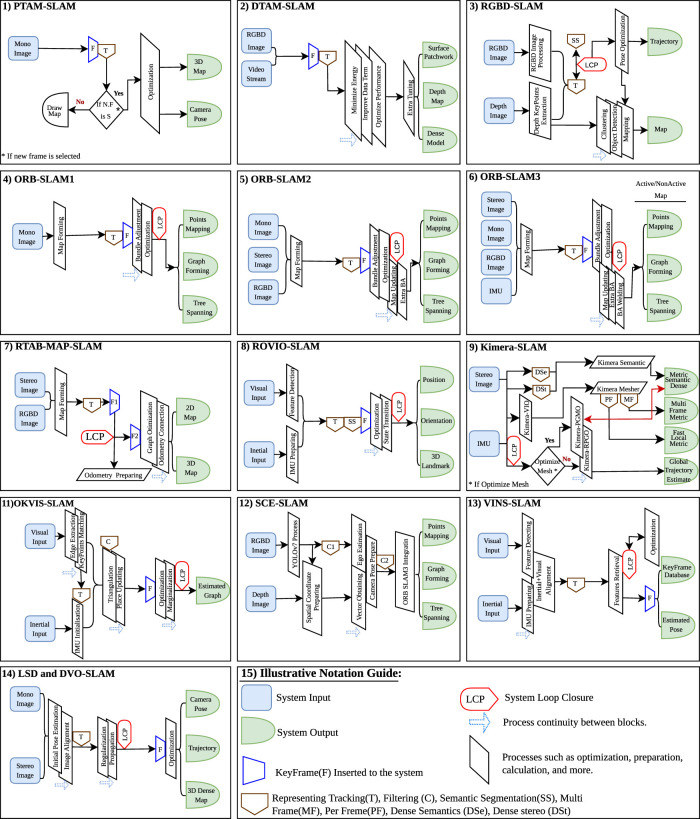
Visual SLAM methods, illustrating the state-of-the-art method and workflow for select notable SLAM methods featured in this study, presented in a simplified view.

The landscape of V-SLAM is composed of a variety of methodologies, which can be divided into three categories, namely, only visual SLAM, visual-inertial SLAM, and RGB-D SLAM (Macario Barros et al., 2022; Theodorou et al., 2022), as shown in Figure 4. In this section, we provide a brief overview of the current state-of-the-art V-SLAM algorithms and techniques, including their methodology, efficiency, time requirements, and processing capacity, as well as whether they are designed to run on-board or off-board computer systems (Tourani et al., 2022). Additionally, we combine various graphical representations to create a single and comprehensive visual representation of the method workflow, as shown in Figure 5.

### 3.1 Only visual SLAM

It is a SLAM system designed to map the environment around the sensors while simultaneously determining the precise location and orientation of those sensors within their surroundings. It relies entirely on visual data for estimating sensor motion and reconstructing environmental structures (Taketomi et al., 2017).

It uses monocular, RGB-D, and stereo cameras to scan the environment, helping robots map unfamiliar areas easily. This approach has attracted attention in the literature because it is cost-effective, easy to calibrate, and has low power consumption in monocular cameras while also allowing depth estimation and high accuracy in RGB-D and stereo cameras (Macario Barros et al., 2022; Abbad et al., 2023). The methods used in this part can be listed herein.

#### 3.1.1 PTAM-SLAM

PTAM-SLAM, which stands for parallel tracking and mapping (PTAM), is a monocular SLAM used for real-time tracking systems. It has 6-DoF camera tracking, which can be used in small scenes (K. and Mu. (2007). This methodology demonstrates remarkable efficiency in dynamic operational settings, consistently providing high performance even in conditions of frequent and unstable lighting variations (Soliman et al., 2023); see Table 2.

**TABLE 2:** Comparative scenarios for actively used visual SLAM methods.

	Sensor				ILR						
SLAM method	M	S	I	O	W-S	Output usage	Application field	RoLI	T2D	Hardware deployment	S.M
**PTAM** K. and Mu	***✓***	**×**	**×**	**×**	M-H	Pose-estimation 3D mapping	Robotics, AR, and VR	**+++**	**++++**	ODROID-XU4, Intel Quad-Core	GPL (2023)
**DTAM** Ne. et al	***✓***	***✓***	**×**	RGBD	S-I	Textured depth map	Robotics, AR, VR, AGV, and simulators	**++**	**+++**	nvidia.gtx.480.gpu, gpgpu-Processors	Rintar (2023)
**RTAB. M** Labbé	***✓***	***✓***	***✓***	Lidar	L-H	2D and 3D mapping	Robotics, VR, AR, and 3D reconstruction	**+++**	**+++**	Jetson Nano, Intel Core.i5.8th.gen	Introlab (2023)
**ORB.S** Mur-A	***✓***	**×**	**×**	**×**	M-H	Tree-spanning and pose-estimating	Robotics mapping indoor navigation	**++++**	**+++**	Intel Core.i7.4700MQ	raulmur (2023a)
**ORB.S2** Leut et al	***✓***	***✓***	**×**	RGBD	M-H	Point-mapping and keyframe selection	Mobile mapping, robotics, VR, and UAVs	**++++**	**++++**	Intel Core-i7.4790 and RealSense-D435	raulmur (2023b)
**ORB.S3** Ca. et al	***✓***	***✓***	***✓***	fish.e	L-H	2D and 3D-Map and tree-spanning	Robotics, security, and 3D reconstruction	**+++++**	**+++++**	Jetson-tx2, pi.3B + nvidia.geforce	uz.slaml (2023)
**RGBD.S** End et al	**×**	**×**	***✓***	RGBD	L-H	Maps, trajectories and 3D point cloud	3D-scanning, robotics and UAVs	**+++**	**++++**	Intel Core.i9.9900k and Quad Core.cpu.8.GB	felix. (2023)
**SCE.S** Son et al	**×**	***✓***	**×**	RGBD	M-I	Camera pose and Semantic Map	Robotics, AR, and AGV	**++++**	**+++**	nvidia.Jetson.AGX, 512.core.Volta.GPU	None
**OKVIS** Leut et al	***✓***	***✓***	***✓***	**×**	M-H	Graph estimation and feature tracking	Robotics, UAVs, and VR	**++++**	**++++**	Up-Board, ODROID.xu4, and Intel® CoreTM.i7	eth.a (2023a)
**ROVIO** Blo. et al	***✓***	***✓***	***✓***	fish.e	L-H	Position and orientation depth map	Robotics, AR, and self-driving. cars	**+++**	**+++**	ODROID-xu4 and Intel i7-2760QM	eth.a (2023b)
**VINS.M** Qin et al	***✓***	**×**	***✓***	**×**	L-H	Keyframe database pose estimation	Robotics, AR, and VR	**+++**	**+++**	Intel Pentium, Intel Core i7-4790 CPU	hkust.a (2023)
**LSD.S** Eng et al	***✓***	***✓***	**×**	RGBD	L-H	Keyframe selection and 3D mapping	Robotics and self-driving cars	**++++**	**+++++**	fpga.zynq.7020.soc Intel® NUC6i3SYH	CVG, T. U. o. M. (2023)
**DVO.S** Kerl et al	**×**	***✓***	**×**	RGBD	S-I	3D mapping image alignment	Robotics and AR Perception	**+++**	**+++**	Sony Xperia.z1, Intel Xeon E5520	tum.v (2023)
**Kimera.S** Ros. et al	***✓***	***✓***	***✓***	Lidar	M-H	Trajectory estimate semantic mesh	Robotics, UAV, VR, and AGV	**++++**	**+++++**	Not mentioned	MIT.S (2023)

-ILR, illumination and light robustness—evaluates how well each SLAM method responds to varying environmental lighting.

-RoLI, range of light intensity—measures the robot’s ability to operate effectively across a broad spectrum of light intensities, from very dark to very bright.

-T2D, tolerance to directionality—assesses the robot’s capability to function in environments with strong directional light sources, such as spotlights and windows.

-W-S, defines the operational scale and application field of the robot (M, medium; L, large; S, small, H, hybrid, I, indoor).

-S.M, sources and materials—provides links to the source codes used in the method.

-VINS.M.S, VINS-Mono SLAM; M, monocular camera; S, stereo camera; IMU, inertial measurement unit; O, other sensors; fish.e, fish-eye camera; rgbd, RGB-D camera.

The system workflow consists of four sequential stages (Klien and Murray. 2007; Fernández-Moral et al., 2013). Input preparation and system initialization involve processes such as monocular camera translation and rotation to improve image efficiency and clarity (De Croce et al., 2019). The tracking process is carried out, where tasks related to image and video processing are performed to prepare data for subsequent mapping procedures. Following that, the optimization and mapping processes are carried out to prepare the map and reveal the outputs, which include the camera pose and the 3D map used in SLAM operations (Klien and Murray. 2007; Servières et al., 2021). All processes and steps are simplified and demonstrated in Figure 5, part 1.

#### 3.1.2 ORB-SLAM

ORB-SLAM stands for oriented FAST (features from accelerated segment test) and rotated BRIEF (binary robust independent elementary features) SLAM (Tourani et al., 2022). This feature-based detector is applicable in both small and large indoor or outdoor fields (Tourani et al., 2022). Due to its real-time capabilities and high-quality map reconstruction, it is widely used in applications such as the human–robot interaction (HRI) (Mur-Artal et al., 2015), augmented reality, and autonomous navigation (Zhu et al., 2022; Yang et al., 2022). ORB-SLAM is designed to handle robust and unstable motion clutter, covering essential processes such as tracking, mapping, and loop closing (Campos et al., 2021). Compared to other advanced V-SLAM methods, ORB-SLAM outperforms by enhancing the dynamic, size, and traceability of the map. It achieves real-time global localization from wide baselines, performs camera re-localization from various viewpoints, and makes better selections for frames and points in the reconstruction process (Ragot et al., 2019; Mur-A and Tars, 2014); see Table 2.

ORB-SLAM1 categorized to be only-visual (Mur-Artal et al., 2015; Mur-A and Tars, 2014), while ORB-SLAM2 expands to both only-visual and RGB-D SLAM (Ragot et al., 2019; Mur-Artal and Tardós 2017a). Furthermore, ORB-SLAM3 furthers its classification to include all three categories: only-visual, visual-inertial, and RGB-D SLAM. This expansion underscores the adaptability and versatility of ORB-SLAM in real-life applications (Zang et al., 2023; Ca et al., 2021; Campos et al., 2021).

The ORB-SLAM methodology process goes through four sequential phases (Mur-Artal et al., 2015; Mur-Artal and Tardós 2017a; Ca et al., 2021). The initial phase involves the sensor input and the tracking process (Joo et al., 2020). Across all ORB-SLAM versions, this phase shares a common approach, focusing on pose preparation and frame generation to facilitate decision-making (Sun et al., 2017). However, the difference lies in input usage; for example, ORB-SLAM1 uses one input, ORB-SLAM2 uses three, and ORB-SLAM3 uses four (Campos et al., 2021). Therefore, the quality and efficiency of the next operation depend on the input in the first stage. In the next phase, local mapping is done by adding new keyframes and creating map points with the localization process simultaneously (Ca et al., 2021). This part remains consistent across all versions, but version 3 enhances its functionality by incorporating additional bundle adjustment for improved feature detection and matching (Dai et al., 2021). The subsequent phase involves loop closing, process optimization, and selecting similar candidate data in all versions. However, versions 2 and 3 include additional steps such as bundle adjustment welding and map merging (Mur-Artal and Tardós, 2017a; Zang et al., 2023). The last stage is preparing the output, focusing on creating the final map that includes essential information such as graphs, lines, point mapping, and 2D and 3D maps for use in the SLAM process (Acosta-Amaya et al., 2023). Figure 5 parts 4, 5, and 6 give a detailed observation about the methods of ORB-SLAM 1, 2, and 3 versions, respectively, showcasing their features and functionalities for a better understanding.

#### 3.1.3 LSD-SLAM

LSD-SLAM, which stands for large-scale direct monocular SLAM, is an advanced technique made for real-time mapping and positioning. It can utilize various camera setups. It is designed for large-scale mapping jobs where it can create a very accurate and detailed map of the working fields. In addition, it stays accurate even with a lower image resolution (Engel et al., 2015; Fernández-Moral et al., 2013). This flexibility makes it a better choice for operating in complex, wide-ranging and dynamic environments and is used in various applications such as robotics and self-driving cars (Mur-Artal et al., 2015; Eng et al., 2014); see Table 2.

LSD-SLAM distinguishes itself from the DTAM-SLAM approach by focusing on areas with strong intensity changes, leaving out regions with little or no texture details. This choice comes from the challenge of figuring out how far things are in areas where there is not much texture inside images. As a result, LSD-SLAM goes beyond what DTAM can do by concentrating on places with strong changes in brightness and ignoring areas with very little texture (Acosta-Amaya et al., 2023; Khoyani and Amini, 2023).

LSD and DVO-SLAM processes can function similarly, and their workflow is structured in five stages (Macario Barros et al., 2022; Luo et al., 2021; Schöps et al., 2014; Engel et al., 2015). The first stage includes inputting mono- and stereo data and preparing them for the next processing step. The second stage is designed for tracking and estimating the initial pose by aligning images from both mono and stereo cameras. The third stage is dedicated to loop closure processes, involving keyframe preparation, regularization, and data updates to prepare frames for subsequent stages. The fourth stage carries out map optimization, including two critical phases, which are direct mapping and feature-based mapping. It also covers processes such as activation, marginalization, and direct bundle adjustment. These operations shape the necessary map and manage its pointsassesses their performance under varyin with semi-dense adjustments for use in the output stage. In the final stage, the estimated camera trajectory and pose with the dense 3D map are prepared for application in robotics’ SLAM functions; see Figure 5, part 14 for a detailed workflow.

#### 3.1.4 DVO-SLAM

DVO-SLAM, which stands for dense visual odometry SLAM, is designed to facilitate real-time motion estimation and map creation using depth-sensing devices, such as stereo and mono cameras (Schöps et al., 2014). It stands out for its ability to generate detailed and accurate environment maps while tracking the position and orientation (Luo et al., 2021; Zhu et al., 2022). DVO-SLAM uses point-to-plane metrics in photo metric bundle adjustment (PBA), enhancing the navigation of robotic systems, especially in situations with less textured points. The point-to-plane metric is a cost function and optimization tool that is used to optimize the depth sensor poses and plane parameters for 3D reconstruction (Alismail et al., 2017; Zhou et al., 2020; Newcombe et al., 2011). These features make DVO-SLAM suitable for more accurate applications such as in robotics and augmented reality (AR), and it is robust for operating in slightly unstable light sources (Khoyani and Amini, 2023; Kerl et al., 2013); see Table 2.

### 3.2 Visual-inertial SLAM

VI-SLAM is a technique that combines the capabilities of visual sensors, such as stereo cameras, and inertial measurement sensors (IMUs) to achieve its SLAM objectives and operations (Servières et al., 2021; Leut et al., 2015). This hybrid approach allows a comprehensive modeling of the environment, where robots operate (Zhang et al., 2023). It can be applied to various real-world applications, such as drones and mobile robotics (Taketomi et al., 2017). The integration of IMU data enhances and augments the information available for environment modeling, resulting in improved accuracy and reduced errors within the system’s functioning (Macario Barros et al., 2022; Mur-Artal and Tardós 2017b). The methods and algorithms used in this approach, while implemented in real-life applications, can be listed as shown in the following section.

#### 3.2.1 OKVIS-SLAM

OKVIS-SLAM, which stands for open keyframe-based visual-inertial SLAM, is designed for robotics and computer vision applications that require real-time 3D reconstruction, object tracking, and position estimation (Kasyanov et al., 2017). It combines visual and inertial measurements to accurately predict the position and orientation of a robot simultaneously (Leut et al., 2015).

It accurately tracks the camera’s position and orientation in real-time control during a robot’s motion (Leutenegger, 2022). It uses image retrieval to connect keyframes in the SLAM pose-graph, aided by the pose estimator for locations beyond the optimization window of visual–inertial odometry (Kasyanov et al., 2017; Wang et al., 2023). For portability, a lightweight semantic segmentation CNN is used to remove dynamic objects during navigation (Leutenegger, 2022). OKVIS’s real-time precision and resilience make it suitable for various applications, including robotics and unmanned aerial vehicles (UAVs). It can operate effectively in complex and unstable illumination environments (Wang et al., 2023); see Table 2.

We have structured the OKVIS-SLAM workflow into three key phases (Leutenegger, 2022; Kasyanov et al., 2017; Wang et al., 2023). The first phase focuses on receiving initial sensor inputs, including IMU and visual data. It initializes the system, conducts IMU integration, and employs tracking techniques to prepare the data for subsequent processing. The second phase is the real-time estimator and odometry filtering phase, covering various operations, such as landmark triangulation and status updating. The triangulation process is used for estimation used to generate the 3D position of visual landmarks to enhance SLAM operation (Yousif et al., 2015). In the last phase, optimization and full graph estimation are performed. This includes loop closure detection, window sliding, and marginalization. The phase selects relevant frames and optimizes the overall graph structure, ultimately providing essential outputs for the SLAM system; see Figure 5, part 11.

#### 3.2.2 ROVIO-SLAM

ROVIO-SLAM, which stands for robust visual-inertial odometry SLAM, is a cutting-edge sensor fusion method that smoothly combines visual and inertial data. This integration significantly enhances navigation accuracy, leading to improved work efficiency in robotics systems (Blo et al., 2015; Wang et al., 2023). It brings valuable attributes for robotics, excelling in robust performance in challenging environments, and presents a smooth interaction between the robot and its surroundings (Li et al., 2023a). It efficiently handles extensive mapping processes, making it suitable for large-scale applications (Kasyanov et al., 2017). Moreover, it operates with low computational demands and high robustness to light, making it ideal for cost-effective robotic platforms designed for sustained, long-term operations (Leutenegger, 2022).

ROVIO-SLAM workflow is divided into three stages (Picard et al., 2023; Nguyen et al., 2020; Schneider et al., 2018). First, data from visual cameras and IMU are obtained and prepared for processing. In the next stage, feature detection, tracking, and semantic segmentation are done for visual data, while IMU data are prepared for integration from the other side. The processing stage involves loop closure operations, new keyframes insertion, and state transition, along with data filtering. State transitions lead to the generation of the key output, which is then transferred to the final stage, providing estimated position, orientation, and 3D landmarks; see Figure 5, part 8.

#### 3.2.3 VINS Mono-SLAM

VINS Mono-SLAM, which stands for the visual-inertial navigation system, is an advanced sensor fusion technology that precisely tracks the motion and position of a robot or sensor in real-time. Utilizing only a single camera and an IMU, it combines visual and inertial data to enhance accuracy and ensure precise functionality of robot operations (Mur-Artal and Tardós, 2017b). Known for its efficiency in creating maps and minimizing drift errors, VINS-Mono excels in navigating challenging environments with dynamic obstacles (Bruno and Colombini, 2021). Its smooth performance in difficult lighting conditions highlights its reliability, ensuring optimal functionality for mobile robots operating in unstable lighting conditions (Song et al., 2022; Kuang et al., 2022). This power-efficient, real-time monocular VIO method is suitable for visual SLAM applications in robotics, virtual reality, and augmented reality (Gu et al., 2022); see Table 2.

The VINS-Mono SLAM workflow is organized into four stages (Qin et al., 2018; Xu et al., 2021). In the first stage, we gathered visual and inertial data and prepared them for acquisition and measurement processing, including feature extraction, matching, and IMU data preparation, and sent them for visual and inertial alignment. The second stage handles loop closure operations and re-localization to adjust old states with additional feature retrieval for the next step. The third stage focuses on process optimization, incorporating bundle adjustments and additional propagation for efficiency. The final stage outputs the system’s estimated pose and a keyframe database, applicable to SLAM; see Figure 5, part 13.

#### 3.2.4 Kimera-SLAM

Kimera-SLAM is an open-source SLAM technique applied for real-time metric semantic purposes. Its framework is highly dependent on previous methodologies such as ORB-SLAM, VINS-Mono SLAM, OKVIS, and ROVIO-SLAM (Ros. et al., 2020). Exhibiting robustness in dynamic scenes, particularly in the presence of moving objects (Wang et al., 2022), Kimera-SLAM showcases resilience to variations in lighting conditions. It operates effectively in both indoor and outdoor settings, making it highly compatible with integration into interactive robotic systems (Rosinol et al., 2021). In summary, Kimera-SLAM provides a thorough and efficient solution for real-time metric-semantic SLAM, prioritizing accuracy, modality, and robustness in its operations (Rosinol et al., 2021); see Table 2.

The procedural workflow of this technique can be summarized in five stages (Ros et al. (2020). First, the input pre-processing includes dense 2D semantics, dense stereo, and Kimera-VIO. It also includes front-end and back-end operations such as tracking, feature extraction, and matching, which yield an accurate state estimation. The second stage involves robust pose graph optimization (Kimera-RPGO), tasked with optimization and the formulation of a global trajectory. Subsequently, the third stage features the per-frame and multi-frame 3D mesh generator (Kimera–Mesher), responsible for the execution and generation of 3D meshes representing the environment. The fourth stage introduces semantically annotated 3D meshes (Kimera-Semantics), dedicated to generating 3D meshes with semantic annotations. This stage sets the groundwork for the subsequent and final stage, where the generated 3D meshes are utilized for output visualization, ultimately serving SLAM purposes, as illustrated in Figure 5, part 9.

### 3.3 RGB-D SLAM

RGB-D is an innovative approach that integrates RGB-D cameras with depth sensors to estimate and build models of the environment (Ji et al., 2021; Macario Barros et al., 2022). This technique has found applications in various domains, including robotic navigation and perception (Luo et al., 2021). It demonstrates efficient performance, particularly in well-lit indoor environments, providing valuable insights into the spatial landscape (Dai et al., 2021).

The incorporation of RGB-D cameras and depth sensors enables the system to capture both color and depth information simultaneously. This capability is advantageous in indoor applications, addressing the challenge of dense reconstruction in areas with low-textured surfaces (Zhang et al., 2021b). The objective of RGB-D SLAM is to generate a precise 3D reconstruction for the system surroundings, with a focus on the acquisition of geometric data to build a comprehensive 3D model (Chang et al., 2023). The methods used in this section are listed as follows:

### 3.3.1 RTAB-Map SLAM

RTAB-Map SLAM, which stands for real-time appearance-based mapping, is a visual SLAM technique that works with RGB-D and stereo cameras (Ragot et al., 2019). It is a versatile algorithm that can handle 2D and 3D mapping tasks depending on the sensor and data that are given (Peter et al., 2023; Acosta-Amaya et al., 2023). It integrates RGB-D and stereo data for 3D mapping, enabling the detection of static and dynamic 3D objects in the robot’s environment (Ragot et al., 2019). It is applicable in large outdoor environments where LiDAR rays cannot reflect and manage the field around the robot (Gurel, 2018). Variable lighting and environmental interactions can cause robotic localization and mapping errors. Therefore, RTAB’s robustness and adaptability to changing illumination and scenes enable accurate operation in challenging environments. It can handle large, complex environments and is quickly adaptable to work with multiple cameras or laser rangefinders (Li et al., 2018; Peter et al., 2023). Additionally, the integration of T265 (Intel RealSense Camera) and implementation of ultra-wideband (UWB) (Lin and Yeh, 2022) address robot wheel slippage with drifting error handling, enhancing system efficiency with precise tracking and 3D point cloud generation, as done in Persson et al. (2023); see Table 2.

The RTAB-MAP SLAM method involves a series of steps that enable it to function (Gurel, 2018; Labbé and Michaud, 2019). Initially, the hardware and front-end stage is responsible for tasks such as obtaining data from stereo and RGB-D cameras, generating frames, and integrating sensors. This stage prepares the frames that will be used in the subsequent stage. After the frames have been processed simultaneously with the tracking process, the loop closure is activated to generate the necessary odometry. Subsequently, the keyframes equalization and optimization processes are initiated to improve the quality of the 2D and 3D maps generated for SLAM applications, as shown in Figure 5, part 7.

#### 3.3.2 DTAM-SLAM

DTAM-SLAM, which stands for dense tracking and mapping, is a V-SLAM algorithm specified for real-time camera tracking. It provides robust six degrees of freedom (6 DoF) tracking and facilitates efficient environmental modeling for robotic systems (Ne. et al., 2011; Macario Barros et al., 2022). This approach plays a fundamental role in advancing applications such as robotics, augmented reality, and autonomous navigation, delivering precise tracking and high-quality map reconstruction. Furthermore, it is slightly dynamic with light; thus, it is accurate to operate in high and strong illumination fields (Zhu et al., 2022; Yang et al., 2022); see Table 2.

The DTAM-SLAM workflow is divided into a series of steps, each with its own purpose (Ne et al., 2011; Macario Barros et al., 2022). It begins with the input such as the RGB-D camera, which helps initialize the system work. In the camera tracking and reconstruction stage, the system selects frames and estimates textures on the image. It then accurately tracks the 6DoF camera motion, determining its exact position and orientation. Furthermore, the optimization framework is activated and uses techniques such as spatially regularized energy minimization to enhance data terms, thereby improving the image quality that is captured from video streaming. As a result, the advanced process tuning carries out operations that improve the method’s performance and producing precise outputs such as dense models, surface patchwork, and texture depth maps (see Figure 5, part 2).

#### 3.3.3 RGBD-SLAM

RGDB-SLAM, which stands for simultaneous localization and mapping using red–green–blue and depth data, is an important method that creates a comprehensive 3D map containing both static and dynamic elements (Ji et al., 2021). This method involves the tracking of trajectories and mapping of points associated with moving objects (Steinbrücker et al., 2011; Niu et al., 2019). Using these data types enhances and provides precise SLAM results (End et al., 2012; Li Q. et al., 2022a). It has the ability to create registered point clouds or OctoMaps for the purpose that can be used for robotic systems (Zhang and Li 2023; Ren et al., 2022). In robotics applications, RGB-D SLAM, specifically V-SLAM, excels in both robustness and accuracy. It effectively addresses challenges such as working in a dynamic environment (Steinbrücker et al., 2011; Niu et al., 2019). The implementation of RGB-D SLAM faced a challenge in balancing segmentation accuracy, system load, and the number of detected classes from images. This challenge was tackled using TensorRT, optimized by YOLOX for high-precision real-time object recognition (Chang et al., 2023; Martínez-Otzeta et al., 2022). It has versatile applications in real-world robotics scenarios, including autonomous driving cars, mobile robotics, and augmented reality (Zhang and Li, 2023; Bahraini et al., 2018); see Table 2.

The RGB-D SLAM workflow can be organized into five essential stages, each playing a crucial role in the SLAM process (Ji et al., 2021; Hastürk and Erkmen, 2021; End et al., 2012). The initial stage involves data acquisition, where RGB-D and depth camera data are collected as the foundational input for subsequent stages. Moving on to the second stage, processing of RGB-D details was activated. During this phase, tasks include feature extraction and pairwise matching while simultaneously addressing depth-related activities, such as storing point clouds, and aligning lines or shapes. In the third stage, activities such as noise removal and semantic segmentation (SS), in addition to loop closure detection, are performed to lay the groundwork for map construction. The fourth stage is dedicated to focus on pose estimation and optimization techniques, leading to improvement in the accuracy of the system output. The final stage involves generating trajectory estimation and maps, refining the outputs for use in SLAM applications in robotic systems; see Figure 5, part 3.

#### 3.3.4 SCE-SLAM

SCE-SLAM, which stands for spatial coordinate errors SLAM, represents an innovative real-time semantic RGB-D SLAM technique. It has been developed to tackle the constraints posed by traditional SLAM systems when operating in dynamic environments (Li et al., 2020). The method was improved to increase the performance of existing V-SLAM methods such as ORB-SLAM3 and makes it useful with greater accuracy and robustness in dynamic situations with the help of merging semantic and geometric data and leveraging YOLOv7 for quick object recognition (Wu et al., 2022). Thanks to these improvements, the SLAM algorithms can be well-suited for dynamic scenarios which allows in greater adaptability and comprehension of system surroundings. This enables robotic systems to operate in more complex circumstances with the fewer mistakes or slippage errors (Liu and Miura, 2021). Moreover, robots equipped with SCE-SLAM are empowered to operate in a more flexible and error-reduced manner, and it can operate in challenging light environments (Son et al., 2023; Ren et al., 2022); see Table 2.

The SCE-SLAM workflow is divided into three key stages (Son et al., 2023). The first stage involves the semantic module. This module processes camera input data and employs Yolov2 to remove noise from the input. The second stage is the geometry module, where depth image analysis and spatial coordinate recovery are performed, preparing the system for integration with ORB-SLAM3. The final stage is dedicated to the integration of ORB-SLAM3. This integration facilitates the execution of processes within ORB-SLAM3. The process works in parallel with the loop closure technique, which results in a more accurate and precise system output; see Figure 5, Part 12.

## 4 Visual SLAM evolution and datasets

The roots of SLAM can be traced back to nearly three decades ago, when it was first introduced by Smith et al. Picard et al. (2023); Khoyani and Amini (2023). Recently, visual SLAM has changed a lot and made a big impact on robotics and computer vision (Khoyani and Amini, 2023). Along this journey, different V-SLAM methods have been created to tackle specific challenges in robot navigation, mapping, and understanding the surroundings (Aloui et al., 2022; Sun et al., 2017). To verify and compare these V-SLAM methods, important datasets have been created which played a crucial role in the field (Pal et al., 2022; Tian et al., 2023a). In this section, we explore the evolution of V-SLAM methods over time and how they have advanced with the help of using the suitable datasets.

**FIGURE 6:**
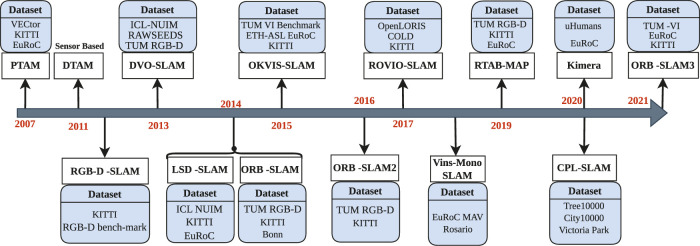
Timeline illustrates the evolutionary journey of SLAM techniques, accompanied by the datasets that have played a pivotal role in their development. It showcases the dynamic progression of SLAM technologies over time, reflecting the symbiotic relationship between innovative methods and the rich variety of datasets they have been tested and refined with.

To offer a more comprehensible perspective, we provide an illustrative timeline depicting the evolution of the most well-known V-SLAM methods, as shown in Figure 6. This graphical representation illustrates the development of the V-SLAM methodologies from 2007 to 2021. These methods have been applied in various fields, including agriculture, healthcare, and industrial sectors, with a specific focus on interactive mobile robots. Additionally, we highlight several significant and widely recognized benchmark datasets crucial to V-SLAM, as shown in the following section.

### 4.1 TUM RGB-D dataset

The TUM RGB-D dataset is a widely used resource in the field of V-SLAM, which helps demonstrate the effectiveness and practicality of V-SLAM techniques. This dataset provides both RGB images and depth maps, with the RGB images saved in a 640 × 480 8-bit format and the depth maps in a 640 × 480 16-bit monochrome (Chu et al., 2018). It offers RGB-D data, making it appropriate for both depth-based and V-SLAM techniques. Its usefulness extends to essential tasks such as mapping and odometry, providing researchers with a considerable volume of data for testing SLAM algorithms across diverse robotic applications (Ji et al., 2021; End et al., 2012). The adaptability of these datasets is remarkable, as they find application in mobile robotics and handheld platforms, demonstrating effectiveness in both indoor and outdoor environments (Martínez-Otzeta et al., 2022; Son et al., 2023).

Some of the recent studies used TUM datasets, such as in Li et al. (2023c). They have leveraged the TUM RGB-D dataset to establish benchmarks customized to their specific research objectives. The study initiated its investigations with RGB-D images and ground truth poses provided by the TUM datasets, utilizing them to construct 3D scenes characterized with real space features. The integrative role assumed by the TUM RGB-D dataset in this context attains profound significance as a fundamental resource within the domain of V-SLAM research. For more details, refer to the TUM RGB-D SLAM dataset.

### 4.2 EuRoC MAV benchmark dataset

The EuRoC MAV benchmark dataset is specifically designed for micro aerial vehicles (MAVs) and contributes a valuable resource in the domain of MAV-SLAM research since it includes sensor data such as IMU and visual data such as stereo images. These datasets, published in early 2016, are made accessible for research purposes and offer a diverse usability in indoor and outdoor applications. Consequently, it serves as a relevant choice for evaluating MAV navigation and mapping algorithms, particularly in conjunction with various visual V-SLAM methodologies (Sharafutdinov et al., 2023; Leutenegger, 2022; Burri et al., 2016).

The EuRoC MAV benchmark dataset, of notable benefits to robotics, is particularly valuable for researchers working on visual-inertial localization algorithms like OpenVINS (Geneva et al., 2020; Sumikura et al., 2019) and ORB-SLAM2 (Mur-Artal and Tardós, 2017a). This dataset incorporates synchronized stereo images, IMU measurements, and precise ground truth data, providing comprehensive resources for algorithm development. Its comprehensive data structure makes it highly suitable for thoroughly testing and validating algorithms tailored for MAV purposes (Burri et al., 2016). For more details, refer to the EuRoC MAV dataset.

### 4.3 KITTI dataset

The KITTI dataset is a widely utilized resource in robotics navigation and SLAM, with a particular emphasis on V-SLAM. Designed for outdoor SLAM applications in urban environments, KITTI integrates data from multiple sensors, including depth cameras, lidar, GPS, and inertial measurement unit (IMU), contributing to the delivery of precise results for robotic applications (Geiger et al., 2013). Its versatility extends to supporting diverse research objectives such as 3D object detection, semantic segmentation, moving object detection, visual odometry, and road-detection algorithms (Wang et al., 2023; Raikwar et al., 2023).

As a valuable asset, researchers routinely rely on the KITTI dataset to evaluate the effectiveness of V-SLAM techniques in real-time tracking scenarios. In addition, it serves as an essential tool for researchers and developers engaged in the domains of self-driving cars and mobile robotics (Geiger et al., 2012; Ortega-Gomez et al., 2023). Furthermore, its adaptability facilitates the evaluation of sensor configurations, thereby contributing to the refinement and assessment of algorithms crucial to these fields Geiger et al. (2013). For more details, refer to the KITTI Vision Benchmark Suite.

### 4.4 Bonn RGB-D dynamic dataset

The Bonn dataset is purposefully designed for RGB-D SLAM, containing dynamic sequences of objects. It showcases RGB-D data accompanied by a 3D point cloud representing the dynamic environment, which has the same format as TUM RGB-D datasets (Palazzolo et al., 2019). It covers both indoor and outdoor scenarios, extending beyond the boundaries of controlled environments. It proves valuable for developing and evaluating algorithms related to tasks such as robot navigation, object recognition, and scene understanding. Significantly, this dataset is versatile enough to address the complexities of applications used in light-challenging areas (Soares et al., 2021; Ji et al., 2021). In addition, it proves to be an important resource for evaluating V-SLAM techniques characterized by high dynamism and crowds where the robot might face the challenge of object detection and interaction with the surrounding environment (Dai et al., 2021; Yan et al., 2022). For more details, refer to the Bonn RGB-D dynamic dataset.

### 4.5 ICL-NUIM dataset

It is a benchmark dataset which is designed for RGB-D applications, serving as a valuable tool for evaluating RGB-D, visual odometry, and V-SLAM algorithms, particularly in indoor situations (Handa et al., 2014). It includes 3D sensor data and ground truth poses, facilitating the benchmarking of techniques related to mapping, localization, and object detection in the domain of robotic systems. Its pre-rendered sequences, scripts for generating test data, and standardized data formats are beneficial for researchers in evaluating and improving their SLAM algorithms (Chen et al., 2020). A unique aspect of the ICL-NUIM dataset is its inclusion of a three-dimensional model. This feature empowers researchers to explore and devise new scenarios for robotic systems, which operates in unknown environments. Moreover, it promotes improvements in V-SLAM, which makes it possible to generate semantic maps that improve robots’ flexibility and adaptability to integration into that environment easily and flexibly (Zhang et al., 2021a). For more details, refer to the ICL-NUIM dataset.

## 5 Guidelines for evaluating and selecting visual SLAM methods

Choosing the right visual SLAM algorithm is crucial for building an effective SLAM system. With the continuous advancements in V-SLAM methodologies responding to diverse challenges, it is essential to navigate structured criteria to deploy and implement precise solutions (Placed et al., 2023; Sousa et al., 2023). In the context of robotic systems, we provide important parameters. We outline them by offering concise explanations of the selection criteria that guide how to choose suitable SLAM methods for field applications. These parameters are listed below.

### 5.1 Robustness and accuracy

When choosing among V-SLAM methods, a key consideration is the robustness and accuracy of the method (Zhu et al., 2022). In particular, a robust algorithm can handle sensor noise, obstacles, and changing environments to ensure continuous and reliable operation (Bongard, 2008). Additionally, accuracy is equally important for creating precise maps and localization, allowing the robot to make informed decisions and move through the environment without errors (Kucner et al., 2023; Nakamura et al., 2023). These qualities collectively enhance the algorithm’s reliability in challenging real-world situations, making them crucial factors for successful mobile robotic applications.

### 5.2 Computational efficiency and real-time requirements

In the application of mobile robotics, the selection of the SLAM algorithm is extremely important, focusing on the efficiency of the process happening inside the robot’s computational architecture (Macario Barros et al., 2022). Therefore, the chosen V-SLAM algorithm must be carefully tailored to meet the computational demands imposed by the real-time constraints of the robot. This entails a delicate balancing act as the selected algorithm should be seamlessly integrated with the available processing power and hardware resources, all while satisfying the stringent real-time requirements of the application. The critical consideration for this step is the quality of the sensors, the professors, and/or computers so that they can generate a quick response and accurate localization in a very limited time (Henein et al., 2020).

### 5.3 Flexible hardware integration

In robotic applications, it is important for researchers to choose a SLAM algorithm that works well with the robot’s sensors. Integrating suitable hardware improves speed and performance in SLAM systems through accelerators, method optimization, and energy-efficient designs (Eyvazpour et al., 2023). Various V-SLAM algorithms are designed for specific sensor types such as RGB-D, lidar, and stereo cameras. This facilitates seamless integration into the SLAM system, enhancing the functionality of utilizing integrated hardware (Wang et al., 2022). Moreover, the availability of ROS packages and open-source software for sensors and cameras provides increased modality and flexibility during system installation. This, in turn, enhances adaptability and makes integration easy and free of challenges (Sharafutdinov et al., 2023; Roch et al., 2023). For example, the OAK-D Camera, also known as the OpenCV AI Kit, is a smart camera that is great for indoor use. It can automatically process data files and use neural reasoning right inside the camera, without needing extra computer power from the robot. This means it can run neural network models without making the robot’s operating system work harder (Han et al., 2023).

### 5.4 System scalability

In SLAM algorithms for robotics, scalability is a vital factor to keep in mind during the design of the system Middleware architecture. It enables rapid situational awareness over large areas, supports flexible dense metric-semantic SLAM in multi-robot systems, and facilitates fast map learning in unknown environments (Castro, 2021). This parameter needs to evaluate the algorithm’s capability to adjust to different mapping sizes and environmental conditions, particularly considering light emission, video, and/or image clarity. It should also provide versatility for various application needs, applicable to both indoor and outdoor scenarios (Laidlow et al., 2019; Zhang et al., 2023).

### 5.5 Adapting to dynamic environments

The ability of a SLAM algorithm to handle dynamic objects in the environment is an important consideration for robotics systems. This parameter assesses the algorithm’s ability to detect, track, and incorporate dynamic objects and moving obstacles into the mapping process (Lopez et al., 2020). It focuses on the algorithm’s capability to enable the robot to handle these objects effectively and respond quickly during the ongoing SLAM process (Wu et al., 2022). A robust dynamic environment should ensure the algorithm’s ability to adapt and respond in real-time applications. This is crucial for systems operating in environments where changes occur instantaneously, such as in interactive robotics applications (Li et al., 2018).

### 5.6 Open-source availability and community support

When choosing a SLAM algorithm for our project, it is important to observe whether it is open-source and has a community of active users. It is important because it makes it easier to customize and adapt the system according to our needs, benefiting from the experiences of the user community (Khoyani and Amini 2023; Xiao et al., 2019). Additionally, having community support ensures that the algorithm receives updates, bug fixes, and improvements. This enhances the reliability and longevity of the algorithm, making it better equipped to handle challenges during system implementation (Persson et al., 2023).

### 5.7 Map data representation and storage

This parameter focuses on how a SLAM algorithm is represented and manages maps, allowing the researcher to determine its suitability for system hardware implementation. The evaluation includes the chosen method’s map representation, whether it is grid-based, feature-based, or point cloud, helping in assessing the efficiency of storing map information in the robotic system without encountering challenges (Persson et al., 2023; Acosta-Amaya et al., 2023). The selection of map representation influences memory usage and computational demands. It is a critical factor for robotic applications, especially those based on CNN and deep learning approaches (Duan et al., 2019).

In conclusion, we have summarized the preceding details in Table 2, offering a comprehensive overview of various V-SLAM algorithms. This table serves as a valuable resource for informed algorithm selection with comparative details for each method. It offers insights into the sensor capabilities, examining the types of sensors most effectively used by each algorithm and their role in facilitating algorithmic functionality. Moreover, the table underscores the potential application domains of the methods, empowering researchers to align their research objectives with suitable V-SLAM methodologies. The table also classifies algorithms based on their mapping scale distinguishing between small-scale (up to 100 m), medium-scale (up to 500 m), and large-scale (1 km and beyond) mapping capabilities (Tian et al., 2023b; Hong et al., 2021).

It also assesses their performance under varying illumination conditions, classifying algorithms based on their robustness, with categories ranging from the lowest, which represents **(+)** and to the highest which represents **(+++++)**. Additionally, the table categorizes the algorithms based on their range of light intensity (RoLI), which reflects the robot’s ability to operate effectively in diverse lighting conditions, spanning from very dim to extremely bright. Moreover, the tolerance to directionality (T2D) category assesses the algorithm’s ability to function in environments with strong directional light sources, such as spotlights and windows. Collectively, these criteria collectively furnish a valuable resource for researchers seeking to pick the most fitting SLAM approach for their specific research endeavors.

## 6 Conclusion

The study simplifies the evaluation of V-SLAM methods, making it easy to understand their behavior and suitability for robotics applications. It covers various active V-SLAM methods, each with unique strengths, limitations, specialized use cases, and special workflows. It has served as a solid foundation for the proposed research methodology for selection among V-SLAM methods. Throughout the research, it becomes evident that V-SLAM’s evolution is importantly linked to the availability of benchmark datasets, serving as a ground base for method validation. Consequently, the work has laid a strong foundation for understanding the system behavior of the working V-SLAM methods. It explores SLAM techniques that operate in the ROS environment, offering flexibility in simplifying the architecture of robotic systems. The study includes the identification of suitable algorithms and sensor fusion approaches relevant to researchers’ work.

By examining previous studies, we identified the potential benefits of incorporating V-SLAM software tools into the system architecture. Additionally, the integration of hardware tools such as the T265 camera and OAK-D camera emerged as a valuable strategy. This integration has a significant potential in reducing errors during robot navigation, thereby enhancing overall system robustness.
